# 712 Association between administration of hydroxycobalamin and acute kidney injury in patients with burn injury

**DOI:** 10.1093/jbcr/irac012.266

**Published:** 2022-03-23

**Authors:** Marcus Lo, Robert Cartotto, George Ho, Stephanie A Mason

**Affiliations:** Queen's School of Medicine, Kingston, Ontario; Ross Tilley Burn Centre, Sunnybrook HSC, Toronto, Toronto, Ontario; University of Toronto, Toronto, Ontario; Ross Tilley Burn Centre, Sunnybrook Health Sciences Centre, University of Toronto, Toronto, Ontario

## Abstract

**Introduction:**

Hydroxycobalamin (HC) is administered to patients with suspected hydrogen cyanide poisoning. Some reports suggest that this practice may be associated with acute kidney injury (AKI). We aimed to evaluate the association between HC administration and AKI after burn injury.

**Methods:**

We conducted a retrospective matched cohort study. All adults admitted to a regional ABA-verified burn center within 24 hours of injury between 2015-2019 with >10% total body surface area (TBSA) flame burn injury were reviewed. We excluded patients who were palliated within 48 hours of admission, and those with pre-existing renal disease. Patients who received HC, either pre-hospital, in the emergency department, or the burn center, were matched on age (+/-2 years) and %TBSA (+/-5%) to up to 5 patients who did not receive HC. Rates of AKI within 7 days of admission, as defined by KDIGO criteria, were then compared. Secondary outcomes included receipt of renal replacement therapy, death, ventilator days, and discharge disposition.

**Results:**

We identified 10 patients who received HC; these patients were matched to 42 patients who did not receive HC. The median age of the HC group was 57 (IQR 43-66), compared to 65 in the non-HC group (IQR 57-65) (p= 0.98). The median %TBSA burn and %TBSA full thickness burn in the HC group and control was 16.5 (14-19) vs 13.5 (11-18) respectively (p= 0.22) and 3.5 vs 3.5 (0-9.5) respectively (p= 0.93). The incidence of inhalation injury in the HC group (n=8, 80%) was significantly higher than in the non-HC group (n=7, 17%)(p< 0.001). All HC patients were intubated within 24 hours of admission vs. only 48% of controls (p=0.003). The HC group also had significantly higher median SOFA scores on admission (9.5 vs 4, p< 0.001) and had a significantly higher admission creatinine [1.08 (0.95-1.26) vs 0.86 (0.71-1.02) mg/dl), p= 0.02]. The median 24-hour crystalloid fluid administered was 5911 mL/kg/%TBSA in the HC group vs 2774 mL/kg/%TBSA burn in controls (p= 0.004). All patients in the HC group developed AKI (n=10, 100%), compared to 64% (n=27) of non-HC patients (p=0.03). There was no significant difference in rates of death (20% vs 10%, p=0.35), Renal Replacement Therapy (20% vs 7%, p=0.21), discharge disposition, or ventilator days between groups.

**Conclusions:**

We identified a small number of patients who received HC over a 5-year period at our burn center. There was an association between HC administration and development of AKI, but there were no differences in mortality, RRT, or ventilator days. HC administration is rare, and our data suggest that the sickest burn injured patients receive it. Larger studies are required to determine if HC administration is associated with AKI, or whether the increased rate of AKI in our study reflects illness severity.

